# Is surgical lymph node assessment justified for ground glass opacity-dominant lung adenocarcinoma?

**DOI:** 10.3389/fonc.2026.1937616

**Published:** 2026-09-11

**Authors:** Benedikt Niedermaier, Patric Engel, Elizabeth Tong, Henrike Deissner, Michael Allgäuer, Laura V. Klotz, Raffaella Griffo, Marc A. Schneider, Florian Eichhorn, Rajiv Shah, Felix J.F. Herth, Martin E. Eichhorn, Claus-Peter Heußel, Hauke Winter

**Affiliations:** 1 Department of Thoracic Surgery, Thoraxklinik, Heidelberg University Hospital, Heidelberg, Germany; 2 Translational Lung Research Center Heidelberg (TLRC-H), Heidelberg, Germany; 3 Division of Systems Biology of Signal Transduction, German Cancer Research Center (DKFZ), DKFZ-ZMBH Alliance, Heidelberg, Germany; 4 Department of Diagnostic and Interventional Radiology with Nuclear Medicine, Thoraxklinik, Heidelberg University, Heidelberg, Germany; 5 Department of Diagnostic and Interventional Radiology, Heidelberg University Hospital, Heidelberg, Germany; 6 Institute of Pathology, Heidelberg University Hospital, Heidelberg, Germany; 7 Translational Research Unit (STF), Thoraxklinik, Heidelberg University Hospital, Heidelberg, Germany; 8 Department of Thoracic Oncology, Thoraxklinik, Heidelberg University Hospital and National Canter for Tumor Diseases (NCT), NCT Heidelberg, Heidelberg, Germany; 9 Department of Pulmonology and Critical Care Medicine, Thoraxklinik, Heidelberg University Hospital, Heidelberg, Germany

**Keywords:** ground-glass opacity, lung adenocarcinoma, lymph node dissection, occult lymph node metastasis, thoracic surgery

## Abstract

**Introduction:**

The necessity of lymph node dissection (LND) as part of curative-intent surgery for ground-glass opacity (GGO)-dominant lung adenocarcinoma has been called into question, given the favorable prognosis and indolent behavior of these tumors. However, the oncological safety of omitting LND in cases of GGO-dominant lung adenocarcinoma remains controversial, and current guidelines continue to recommend it.

**Methods:**

This study was designed as a retrospective, single-center cohort study. Patients with cT1N0M0 lung adenocarcinoma who underwent surgical resection between 2010 and 2023 were categorized as GGO-dominant or solid-dominant cohorts based on consolidation-to-tumor ratios in preoperative computed tomography scans. The primary endpoint was the incidence of occult lymph node (LN) metastasis.

**Results:**

A total of 726 consecutive patients were included in this study, categorized as GGO-dominant in 50 patients and solid-dominant in 676 patients. GGO-dominant tumors were more frequently observed in female patients (p = 0.03), whereas no significant differences were found between the groups with respect to age, smoking status, or ECOG performance status. GGO-dominant morphology was associated with lower pT stage and lower rates of pleural and lymphovascular invasion. The incidence of occult LN metastasis was 4% (n=2) in the GGO-dominant cohort and 11.8% (n=80) in solid-dominant cohort. Only solid tumor size was a significant predictor of occult LN metastasis in multivariable logistic regression (OR 1.39 per 5mm increase, 95% CI 1.10-1.77, p = 0.006). Survival analysis revealed a higher rate of freedom from recurrence (p = 0.04) and favorable trends in overall survival (p = 0.09) and recurrence-free survival (p = 0.086) in the GGO-dominant cohort.

**Conclusions:**

The hypothesis that GGO-dominant morphology reliably predicts negative lymph node involvement was not confirmed. These findings support the continued role of surgical nodal assessment for accurate pathological staging.

## 1 Introduction

Lung cancer screening programs, which have been implemented in many countries around the world, are expected to lead to an increase in the number of patients diagnosed with stage I disease (1). However, even among patients with stage I lung cancer, radiologic and histologic features of invasiveness may still indicate significant differences in prognosis (2, 3). In lung adenocarcinoma, both the non-invasive lepidic growth pattern in histology and the characteristic of ground-glass opacity (GGO) in computed tomography (CT) represent low-malignant tumors with excellent prognosis, supporting the development of risk-adapted management approaches (2, 4–7). Therefore, recent trials have investigated various aspects of personalized surgical treatment for this group of patients, such as the safety of sublobar resection, the safety of omitting lymph node dissection (LND), or the appropriateness of active surveillance strategies versus surgical intervention (8–12).

In contrast to lepidic histology, GGO appearance has proved to be the more pragmatic parameter for clinical decision-making, since there is currently no reliable method to predict the proportion or predominance of lepidic growth from preoperative imaging or core biopsy (5, 13). To quantify the proportion of GGO, lesions can be classified by consolidation-to-tumor ratio (CTR), using the solid component’s maximum diameter divided by the total lesion’s maximum diameter (14). In the JCOG0201 trial, lesions with less than or equal to 3 cm in size with a CTR of 0.5 or less (GGO-dominant lesions) were defined as “radiological non-invasive” (15). These limits have since been widely accepted to define a low-risk patient population in clinical trials evaluating risk-adapted treatment strategies (12, 16). Specifically, the practice of performing a systematic LND on every patient undergoing curative-intent surgery for early-stage lung cancer has been called into question, as it may not be oncologically necessary and omitting LND can reduce postoperative complications (8, 17–19). While an increasing number of studies suggest that LND may be safely omitted in selected patients with GGO-dominant tumors undergoing pulmonary resection, systematic LND with histopathological assessment of lymph nodes (LN) remains the standard approach for pathological staging and is endorsed by current international guidelines (20–23). Nevertheless, the external validity of the existing evidence is limited by the predominance of studies from selected geographic regions and relatively homogeneous ethnic populations.

The proposed omission of LND is predicated on the assumption that occult LN metastases are absent or exceedingly rare in carefully selected patients (8, 24). However, before this approach can be adopted more widely, its safety must be confirmed in diverse patient populations and healthcare settings, given potential geographic and ethnic differences in tumor epidemiology, biology, and pathology. Against this background, the present study was designed to investigate the incidence of occult LN metastases in GGO-dominant and solid-dominant lung adenocarcinomas.

## 2 Methods

### 2.1 Trial design and patients

This study was a retrospective, single-center cohort study designed to evaluate the necessity of LND (the current standard of care in curative-intent lung cancer surgery), in patients with GGO-dominant invasive lung adenocarcinoma. Patients with cT1N0M0 invasive lung adenocarcinoma who underwent complete resection between 01/2010 and 12/2023 were considered for inclusion in the study and stratified according to CTR, classifying tumors with less than or equal to 0.5 as GGO-dominant and > 0.5 as solid-dominant on the basis of preoperative CT images. CTR was calculated as ratio of the maximum diameter of the solid component to maximum diameter of the entire tumor (including the GGO and solid components). Exclusion criteria were missing preoperative CT images, multiple synchronous lung tumors, incomplete resection, minimally-invasive adenocarcinoma (MIA) or *in-situ* adenocarcinoma (AIS), or if no LND was performed during surgery. The study was approved by the ethics committee of Heidelberg University (S-174/2019) and adhered to the Declaration of Helsinki for ethics in medical research.

### 2.2 Assessments and endpoints

Demographic and pathologic data, CT images and survival data were retrospectively retrieved from the hospital database for further analysis. The preoperative workup in clinical routine consisted of contrast-enhanced chest CT, 18F-fluorodeoxyglucose positron emission tomography (18F-FDG PET) with CT, and brain magnetic resonance imaging (MRI). The LN were classified as uninvolved (cN0) if the short axis of the mediastinal or hilar LN on CT had a diameter of less than 1 cm and if no FDG accumulated in these LN on the 18F-FDG-PET images. A preoperative CT scan was established as a minimum requirement for thoracic staging assessment for inclusion in this study. Invasive LN staging using endobronchial ultrasonography or mediastinoscopy were not routinely performed. Preoperative contrast-enhanced CT scans were generally performed within 6 weeks before surgical resection as part of routine clinical care using multidetector CT scanners according to institutional thoracic imaging protocols. Images were acquired during inspiratory breath-hold and reconstructed using standard lung and mediastinal reconstruction kernels. Tube voltage and tube current were selected according to routine clinical protocols with automatic exposure modulation where available. Because of the retrospective study design and the long inclusion period, CT acquisition parameters were not prospectively standardized. Lung tumors were quantified on the most recent CT scan before curative-intent surgery, using the automated lung lesion segmentation tool in syngo.via (VB60; Siemens Healthineers AG, Erlangen, Germany). GGO was defined as a hazy area of increased attenuation not obscuring the underlying bronchial or vascular structures. The maximum axial diameter of the solid component and the entire tumor including GGO components were segmented separately and manually corrected, if necessary. Measurements were carried out by a research fellow (PE) under the supervision of experienced chest radiologists (ET, CPH). Investigators were blinded to the pathologic stage and survival outcomes of patients.

The study’s primary endpoint was the rate of occult LN metastasis. Surgical LND was performed according to international standards recommending a minimum of at least one hilar N1 station and three mediastinal N2 stations (20–23). The lymph node map of the eighth edition of the TNM classification system was used (25). Histopathologic assessment was carried out in clinical routine at the Institute of Pathology of Heidelberg University Hospital by specialized thoracic pathologists routinely seeing all lung cancer cases. Tumor stage was reported according to the eighth edition of the TNM classification by the Union for International Cancer Control and reclassified as appropriate for patients initially staged according to the 7th edition of the TNM classification (13). Secondary endpoints were overall survival (OS), recurrence-free survival (RFS) and freedom from recurrence (FFR). OS was defined as time from surgery to death from any cause; RFS was defined as time from surgery to recurrence or death from any cause. For FFR analysis, only tumor recurrence was considered an event, while patients were censored at the time of death or last follow-up. To assess recurrence, postoperative surveillance examinations were performed according to standard of care procedure in routine intervals.

### 2.3 Statistical analysis

Demographic, clinical, and pathological characteristics were summarized using descriptive statistics. Continuous variables were reported as medians with interquartile range (IQR) and categorical variables were presented as counts and percentages. Group comparisons were performed using the Mann-Whitney U test for continuous data and Fisher’s exact test for categorical data. The association between CTR and occult LN metastasis was evaluated using univariable and multivariable logistic regression analysis including prespecified variables based on clinical relevance. Multicollinearity was assessed using variance inflation factors (VIFs). The multivariable analysis was performed as a complete-case analysis. Model discrimination was assessed using the area under the receiver operating characteristic curve (AUC). Survival was estimated by the Kaplan-Meier method with the logrank (Mantel-Cox) test. The median follow-up time was calculated using the reversed Kaplan-Meier method. Two-sided p-values of <0.05 were considered statistically significant. Statistical analyses were conducted using R (version 4.5.2, R Foundation for Statistical Computing, Vienna, Austria) and Prism (version 11.0.1, Graphpad Software Inc., Boston, USA).

## 3 Results

### 3.1 Study population

**Figure 1:**
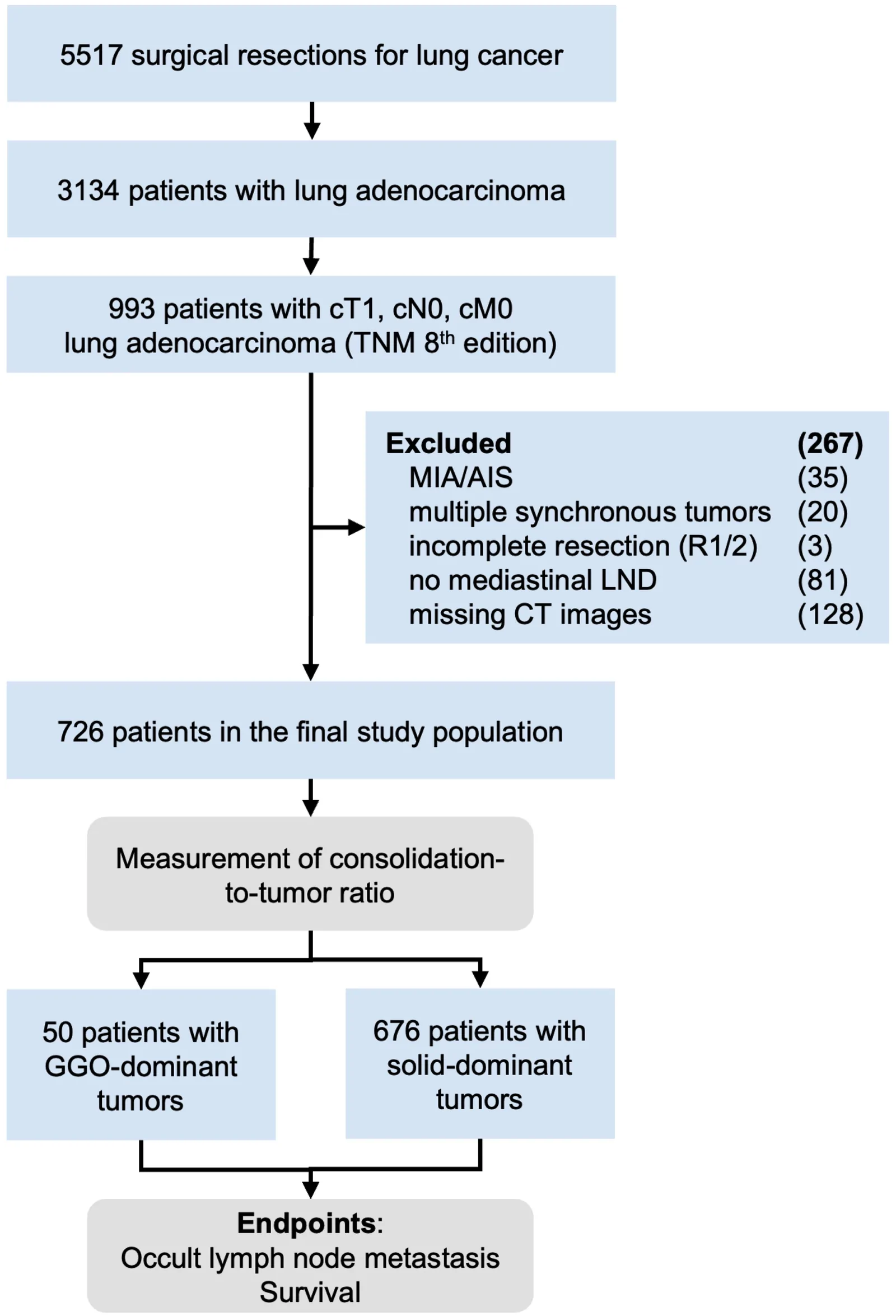
Flow chart showing patient selection and trial design. MIA, minimally invasive adenocarcinoma; AIS, adenocarcinoma *in situ*; LND, lymph node dissection; CT, computed tomography; GGO, ground glass opacity.

**Figure 2:**
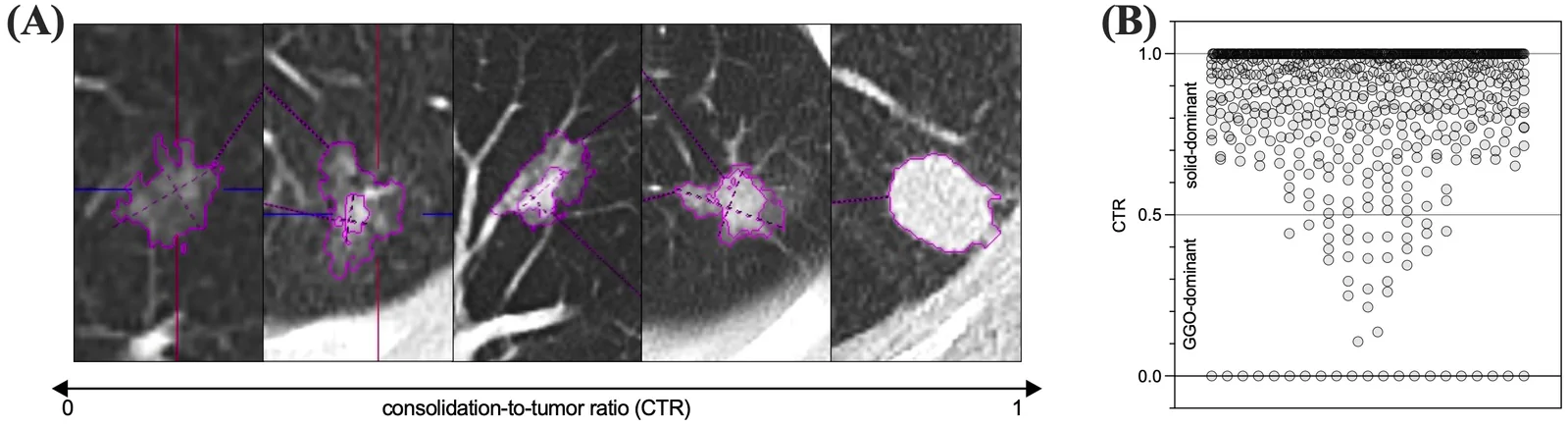
Assessment of consolidation-to-tumor ratio (CTR) on preoperative computed tomography images. **(A)** Representative preoperative chest CT images with measurement of solid and GGO-components in syngo.via. **(B)** Distribution of CTR in the study population. Dots represent individual patients. Patients were classified as GGO-dominant (CTR ≤ 0.5) or solid-dominant (CTR > 0.5).

In this retrospective cohort study, 726 consecutive patients were included according to selection criteria (**Figure 1**). Baseline characteristics are reported in **Table 1**. Tumors were classified as GGO-dominant in 50 patients and solid-dominant in 676 patients according to CTR measurements. While the overall tumor size was similar in both groups (20.25 mm in GGO-dominant vs. 21.15 mm in solid-dominant, p = 0.38), the solid component was significantly smaller in the GGO-dominant cohort (4.9 mm vs. 18.7 mm, p < 0.0001). **Figure 2** shows representative images from the preoperative chest CT scan with corresponding radiological measurements and the distribution of the CTR across the study population. Median age and Easter Cooperative Oncology Group performance status (ECOG PS) were well balanced between the groups at 63 years in the GGO-dominant cohort and 66 years in the solid-dominant cohort (p=0.44). The majority of patients presented in ECOG PS 0 (78.0% and 77.5%, respectively). The proportion of women was higher in the GGO-dominant study cohort (68.0% vs. 50.9%, p = 0.027). Smoking history was comparable between the cohorts with 18% and 15.5% never-smokers in the GGO- and solid-dominant cohorts, respectively (p = 0.21). Tumor location was also well balanced between the cohorts.

**Table 1:** Demographic characteristics.

Variable	GGO-dominant (n=50)	Solid-dominant (n=676)	p-value
Age, years	63 (59-71)	66 (59-72)	0.437
Sex			0.027
Male	16 (32%)	332 (49.1)	
Female	34 (68%)	344 (50.9)	
ECOG-PS			>0.99
0	39 (78%)	524 (77.5%)	
1	11 (22%)	151 (22.3%)	
2	0 (0%)	1 (0.1%)	
Smoking history*			0.209
Never	9 (18%)	98 (15.5%)	
Former	29 (58%)	305 (48.3%)	
Current	12 (24%)	228 (36.1%)	
Tumor location			0.711
RUL	19 (38%)	234 (34.6%)	
RML	2 (4%)	43 (6.4%)	
RLL	7 (14%)	123 (18.2%)	
LUL	16 (32%)	167 (24.7%)	
LLL	6 (12%)	109 (16.1%)	
Total tumor size, mm	20.3 (16 - 25.0)	21.2 (16.7 - 25.5)	0.380
Solid tumor size, mm	4.9 (0.0 - 8.9)	18.7 (14.7 - 23.2)	<0.0001
CTR	0.25 (0.0 - 0.42)	0.96 (0.84 - 1.0)	<0.0001

Data is presented as median (interquartile range) or absolutes (%). Group comparisons were performed using the Mann-Whitney U test for continuous data, and Fisher’s exact test for categorical data. ECOG-PS, Eastern Cooperative Oncology Group performance status; RUL, right upper lobe; RML, right middle lobe; RLL, right lower lobe; LUL, left upper lobe; LLL, left lower lobe; CTR, consolidation-to-tumor ratio; n.s., not significant. *Smoking-status percentages and the p-value were calculated using patients with available smoking-status data only; smoking-status data were missing for 45 patients.

### 3.2 Pathologic outcomes

**Figure 3:**
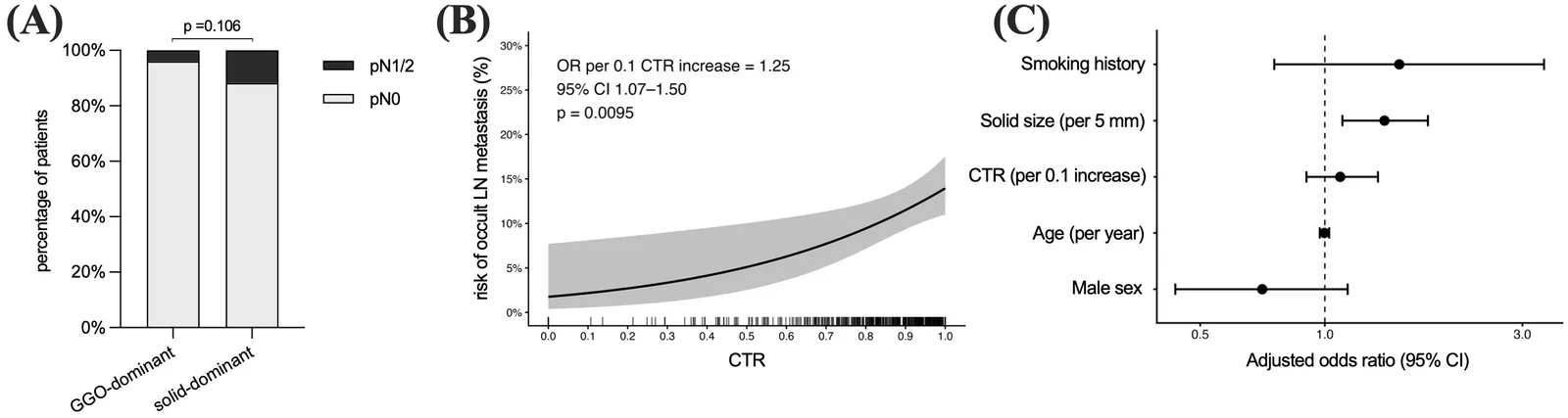
Impact of CTR on the incidence of occult lymph node metastasis. **(A)** Incidence of occult lymph node metastasis in patients with GGO-dominant or solid-dominant lung adenocarcinoma. Group comparison was performed using Fisher’s exact test. **(B)** Predicted probabilities of occult lymph node metastasis and corresponding 95% confidence intervals from fitted univariable logistic regression with rug plot indicating individual patients. **(C)** Forest plot of multivariable logistic regression analysis for predictors of occult lymph node metastasis. Odds ratios and 95% confidence intervals (CI) are shown.

Surgical and pathologic outcomes are shown in **Table 2**. In the GGO-dominant cohort, significantly more patients underwent a sublobar resection (38% vs. 18.3%, p = 0.002). Additionally, the median number of resected LN was lower in the GGO-dominant cohort with 18.5 (IQR, 14-24) vs. 22 (IQR, 16-29) in the solid-dominant cohort (p = 0.02). The median number of resected LN stations was 8 (IQR, 6-9) in the GGO-dominant cohort and 8 (IQR, 7-9) in the solid-dominant cohort. The incidence of occult LN metastasis was 4% (n=2) in patients with GGO-dominant tumors and 11.8% (n=80) in patients with solid-dominant tumors (p = 0.106, **Figure 3a**), corresponding to an absolute risk difference of -7.8% (95% CI, -11.8% to +1.9%), with a relative risk of 0.34 (95% CI, 0.09 to 1.33). This included N1 disease in one (2%) vs. 49 (7.2%) patients, and N2 disease in one (2%) vs. 31 (4.6%) patients, respectively. In the GGO-dominant cohort, occult LN metastases occurred only in patients with part-solid lesions; detailed characteristics of these patients are shown in supplementary **Supplementary Table S1**.

**Table 2:** Outcomes of surgery and pathology.

Variable	GGO-dominant (n=50)	Solid-dominant (n=676)	p-value
Surgical procedure			0.002
Lobectomy	31 (62%)	552 (81.7%)	
Sublobar resection*	19 (38%)	124 (18.3%)	
Total resected lymph nodes	18.5 (14-24)	22 (16-29)	0.021
Resected lymph node stations	8 (6-9)	8 (7-9)	0.056
pN stage			0.106
N0	48 (96%)	596 (88.2%)	
N1/2	2 (4%)	80 (11.8%)	
pT stage			0.015
T1	43 (86%)	481 (71.2%)	
T2	5 (10%)	175 (25.9%)	
T3	1 (2%)	18 (2.7%)	
T4	1 (2%)	2 (0.3%)	
Visceral pleura invasion	5 (10%)	156 (23.1%)	0.033
Lymphatic invasion	1 (2%)	131 (19.4%)	0.001
Vascular invasion	2 (4%)	133 (19.7%)	0.004
Adjuvant therapy			0.568
Yes	2 (4%)	48 (7.1%)	
No	48 (96%)	628 (92.9%)	

Data is presented as median (interquartile range) or absolutes (%). Group comparisons were performed using the Mann-Whitney U test for continuous data and Fisher’s exact test for categorical data. GGO, ground-glass opacity; n.s., not significant. *including both wedge resection and segmentectomy.

In logistic regression analysis, the predicted probability of occult LN metastasis increased continuously across the spectrum of CTR values (OR 1.25 per 0.1 increase in CTR, 95% confidence interval (CI) 1.06-1.47, p = 0.009), indicating a positive association between increasing CTR and nodal involvement (**Figure 3b**). However, only solid tumor size remained a significant predictor of occult LN metastasis in multivariable analysis (OR 1.39 per 5mm increase, 95% CI 1.1-1.77, p = 0.006; **Figure 3c**). The multivariable model included 681 patients with 80 occult LN metastasis events; 45 patients were excluded because of missing smoking-history data. Despite the mathematical relationship between CTR and solid component size, VIFs were low for all model variables (1.01-1.41), indicating no relevant multicollinearity. Model discrimination was modest, with an AUC of 0.655. GGO-dominant morphology was associated with lower T stage and lower rates of vascular, lymphatic, and visceral pleural invasion (**Table 2**).

### 3.3 Survival

**Figure 4:**
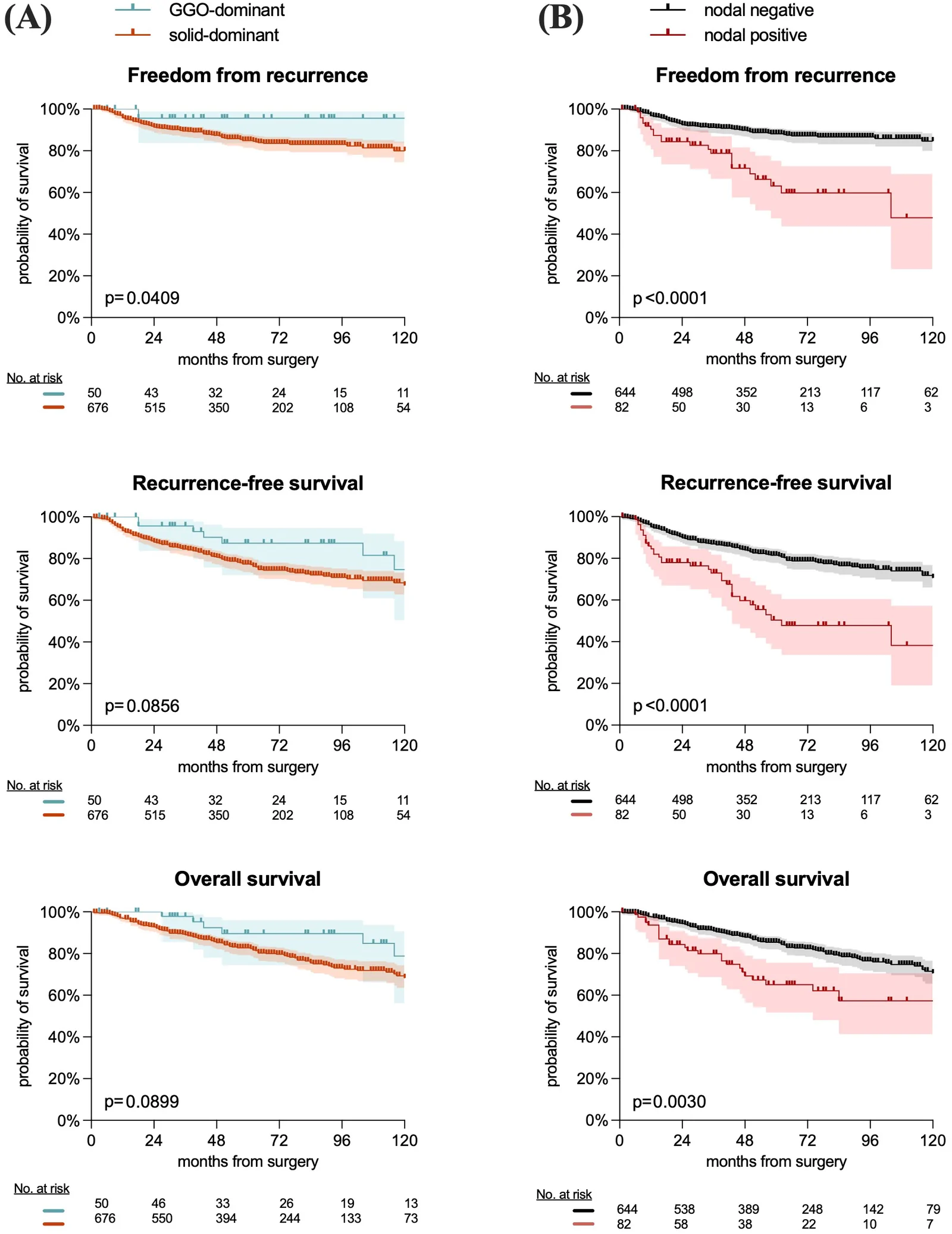
Freedom from recurrence (FFR), recurrence-free survival (RFS) and overall survival (OS). **(A)** FFR, RFS and OS in patients with GGO-dominant or solid-dominant lung adenocarcinoma. **(B)** FFR, RFS and OS in patients with or without occult lymph node metastasis. Kaplan-Meier survival curves are shown with 95% confidence bands. Results of the Log-rank (Mantel-Cox) test are given for individual curve comparison.

The median duration of follow up was 68 months. In total, 89 recurrence events and 152 death events were recorded. In an exploratory unadjusted comparison, the GGO-dominant cohort showed a significantly higher rate of freedom from recurrence (p = 0.041), and trends towards favorable recurrence-free survival (p = 0.086) and overall survival (p = 0.090, **Figure 4a**). The observed FFR was 95.7% (95% CI, 83.7-98.9%) vs. 89.6% (95% CI, 86.7-91.7%) at 3 years and 95.7% (95% CI, 83.7-98.9%) vs. 84.9% (95% CI, 81.4-87.8%) at 5 years for the GGO-dominant and solid-dominant cohorts, respectively. RFS was 95.7% (95% CI, 83.7-98.9%) vs. 84.5% (95% CI, 81.2-87.1%) at 3 years and 87.4% (95% CI, 72.0-94.6%) vs. 77.4% (95% CI, 73.4-80.6%) at 5 years for the GGO-dominant and solid-dominant cohorts, respectively. OS was 97.8% (95% CI, 85.6-99.7%) vs. 88.9% (95% CI, 86.0-91.1) at 3 years and 89.6% (95% CI, 74.4-96.0%) vs. 82.7% (95% CI, 79.1-85.5%) at 5 years. The finding of occult LN metastasis was significantly associated with poor FFR, RFS and OS compared to pN0 stage (**Figure 4b**).

## 4 Discussion

Lymph node dissection is a cornerstone of curative-intent surgery for lung cancer and is currently recommended by major international guidelines (20–23). However, in the subgroup of early-stage lung cancer with a GGO component, the necessity of LND is controversial (8, 10, 26). Here, we retrospectively assessed the incidence of occult LN metastasis in patients with cT1N0M0 lung adenocarcinoma to verify the rationale for LND in GGO-dominant lung adenocarcinoma. Among 50 patients with GGO-dominant lung adenocarcinoma, occult LN metastasis were found in 2 patients (4%), demonstrating that GGO-dominant morphology does not identify a uniformly node-negative population among patients with invasive lung adenocarcinoma. These findings support the continued role of surgical nodal assessment for accurate pathological staging. The observed rate of 4% in the GGO-dominant cohort is in line with previous evidence (27, 28).

The overall association of GGO vs. solid appearance in CT to invasive histologic features and metastatic potential is well described (27–31). Several recent trials reported that GGO-dominant lesions have a lower risk of LN metastasis compared with that of solid-dominant tumors (19, 27, 28). Here, we also showed that the risk of occult LN metastasis rises with increasing CTR. However, multivariable analysis revealed that the size of the solid component is the stronger predictor of LN metastasis, while the impact of CTR did not remain significant after multivariable adjustment. Solid tumor size was repeatedly reported to correlate with greater risk of LN metastasis and might be the better surrogate for clinical decision making compared to CTR (29, 30, 32).

Compared with solid-dominant tumors, the lower incidence of occult LN metastases in the GGO-dominant cohort did not reach statistical significance. However, this finding should be interpreted with caution. The small number of GGO-dominant tumors (n = 50) and the low event rate (2 occult LN metastases) substantially limited statistical power, resulting in wide confidence intervals around the estimated effect. The observed numerical difference (4.0% vs. 11.8%) suggests that larger adequately powered studies are required to more precisely estimate the risk of occult nodal metastasis in GGO-dominant lung adenocarcinoma. Additionally, the reported regression analysis demonstrated a clear trend of decreasing likelihood of occult LN metastasis with lower CTR, supporting the notion that CTR is associated with the risk of nodal spread.

Importantly, reduced nodal assessment may encompass omission of systematic LND with preservation of limited or selective nodal sampling, whereas complete omission of nodal assessment represents a more extensive de-escalation strategy. Our study was not designed to compare these different surgical approaches. Rather, the detection of occult nodal disease demonstrates that complete omission of nodal assessment based on CTR ≤ 0.5 alone carries a risk of leaving nodal metastases pathologically unidentified. Also, this study was not designed to evaluate the impact of LND on survival. Recent prospective evidence suggests that although occult LN metastasis may rarely be identified in patients with GGO-dominant NSCLC, long-term survival after omission of LND is similar, supporting the feasibility of a de-escalation strategy (8, 33, 34). Future studies should establish whether systematic LND can be safely replaced by limited nodal sampling, or whether nodal assessment can be completely omitted in more stringently selected low-risk populations.

Here, we chose the limits of CTR of 0.5 or less to define patient cohorts, because it is a widely accepted stratification (12, 16). However, pure GGOs and part-solid lesions may substantially differ in their risk of lymphatic spread (24, 27, 35). Consequently, recent trials have also explored stricter limits to define radiologic low-risk tumors, either considering only pure GGOs (CTR = 0) or part-solid lesions with a CTR of 0.25 or less. Recently, Catelli et al. reported 0% LN metastasis in a cohort of 400 resected pure GGOs (36). In line with these observations, the two patients in the GGO-dominant cohort that showed occult LN metastases had part-solid lesions, and no LN metastases were observed in patients with pure GGOs here. Although Al Shammari et al. reported a high prevalence of invasive cancer even in pure GGOs, the incidence of LN metastases in this subgroup appears to be exceedingly rare (37). Our results should therefore be interpreted for a broader GGO-dominant population, and cannot directly be generalized to specifically pure GGOs or almost-pure GGOs.

Regarding survival outcomes, our results are in line with the overall consensus that GGO-dominant adenocarcinoma represents an indolent subtype of lung cancer (38). Although the unadjusted comparison between both cohorts may be subject to confounding, we found that patients with GGO-dominant tumors exhibited higher rates of freedom from recurrence after complete resection. The trend of favorable RFS and OS in patients with GGO-dominant lung adenocarcinoma is in accordance with multiple prior studies reporting favorable prognosis associated with GGO-morphology, although it did not reach significance in this study (3, 39). In early-stage tumors with an overall good prognosis, the impact of unrelated death events on OS and RFS can mask the impact of tumor recurrence. Thus, freedom from recurrence is the most direct measure of true differences in oncologic outcomes, considering only tumor recurrence an event. Furthermore, GGO-dominant morphology was inversely associated with known poor prognostic factors like higher pT stage and vascular, lymphatic, or visceral pleural invasion, indicating favorable prognosis in patients with GGO-dominant lung adenocarcinoma. Our finding of poor survival in patients with occult nodal disease corroborates the well-known impact of lymphatic spread on survival and emphasizes the role of surgical LND for prognostic stratification and the indication for adjuvant therapy.

We purposefully excluded pre-invasive lesions like AIS and MIA in this study, because it was repeatedly demonstrated that these do not yet form LN metastasis, and survival after resection is 100% (5, 40–42). Thus, the presented data should be interpreted in context of a pathologically selected cohort of invasive adenocarcinoma, and cannot be generalized to the full spectrum of GGO-dominant tumors including pre-invasive lesions. In contrast to AIS and MIA, risk estimation in invasive stage I lung adenocarcinoma is less unequivocal. We previously reported that even in grade 1, lepidic-predominant tumors, minor high-risk components can already occur in a subgroup of patients and are associated with poor recurrence-free survival (4). Similarly, although GGO-dominant lung adenocarcinoma certainly represents a low-risk subgroup, the incidence of LN metastasis was not zero in this study and could be associated with high-risk features in affected tumors even within GGO-morphology. GGO/lepidic phenotype alone might not be a reliable predictor of indolent tumor behavior in all patients. Further research is warranted to improve risk stratification and identify patients with elevated risk even within this low-risk subgroup.

Interestingly, compared with earlier studies in Asian populations, only a small proportion of patients with lung adenocarcinoma had a GGO-dominant tumor (50 patients, 7% of the total study population), which may indicate significant differences in tumor epidemiology and biology between different regions and continents (3, 12, 39, 43). We previously reported a considerably lower incidence of tumors with lepidic growth in a large German cohort compared to reports from Asia, reiterating this disparity (2, 6, 44). These observations emphasize the need for continued validation across different institutions around the world, to enable accurate and balanced risk assessment for all lung adenocarcinoma patients. We also observed a higher proportion of female patients in the GGO-dominant cohort. Female sex is repeatedly found to be associated with both lepidic histology and GGO morphology (45). The underlying factors driving lung adenocarcinoma development in females remain subject of current research, but are likely associated with a complex interplay of genetic predisposition, epigenetic regulation and immunomodulatory effects (46, 47).

The study is limited by its retrospective design that potentially introduces selection bias and biased surgical procedures, i.e. reflected in more sublobar resections and fewer resected LN in the GGO-dominant cohort. However, with a median of 18.5 resected nodes per patient in the GGO-dominant cohort, we are confident the surgical procedures were adequate to assess the primary endpoint of occult LN metastasis. Furthermore, 81 patients were excluded from the study cohort because no LND was performed intraoperatively, potentially introducing selection bias. The most common reasons for omission of LND in these patients were nodules of uncertain dignity that were only diagnosed as adenocarcinoma on final pathology, as well as intraoperative factors such as cardiopulmonary instability or severe adhesions. The limited sample size and low event number in the GGO-dominant cohort reduces statistical power, especially because the expected incidence of occult LN metastasis is small. The measurement of CTR is not always entirely objective and requires further refinement, although our semi-automated workflow limits variability and radiologic investigators were blinded to the histopathologic outcome to prevent observer bias. Future work should address the reproducibility of radiologic evaluations of CTR. Furthermore, various additional factors that could affect LN metastasis, such as histologic growth patterns and additional PET/CT or CT characteristics, were not investigated. This is because we sought to focus on the representative parameter of solid/GGO proportions as a surrogate for surgical decision-making.

In conclusion, occult lymph node metastases were observed in 4% of patients with GGO-dominant invasive lung adenocarcinoma, demonstrating that CTR ≤ 0.5 alone does not identify a uniformly node-negative population. These findings support the continued role of surgical nodal assessment for accurate pathological staging and the identification of patients potentially eligible for adjuvant therapy. Of note, this study does not establish a direct therapeutic or survival benefit of surgical nodal assessment in GGO-dominant invasive lung adenocarcinoma. Future studies are warranted to identify patients in whom nodal assessment can safely be reduced or omitted and to identify more accurate predictors of LN metastasis, which may inform personalized and risk-adapted LND strategies in lung cancer beyond the GGO/solid distinction.

## Data Availability

The raw data supporting the conclusions of this article will be made available by the authors, without undue reservation.
