# Supplementary material for: Is surgical lymph node assessment justified for ground glass opacity-dominant lung adenocarcinoma?
Source: Front Oncol. 2026 Sep 11;16:1937616. doi: 10.3389/fonc.2026.1937616 (PMC13612291; doi:10.3389/fonc.2026.1937616)
Supplement: Supplementary file 1 [file Table1.docx]

**Supplementary Table S1**

**Patient characteristics of GGO-dominant tumors with occult nodal metastases**

| **#** | **age** | **sex** | **tumor location** | **total tumor size** | **solid tumor size** | **CTR** | **SUVmax** | **surgery** | **TNM** | **number of positive nodes** | | **location of positive nodes** | | **predominant histologic**  **pattern** | **adjuvant therapy** | **follow up** | **recurrence** | **death** |
| --- | --- | --- | --- | --- | --- | --- | --- | --- | --- | --- | --- | --- | --- | --- | --- | --- | --- | --- |
| 1 | 61 | female | LLL | 23.5 mm | 10.1 mm | 0.43 | n/a | lobectomy | pT2a, pN2, L1, V1, Pl1 | 1 | 7 | | acinar | | yes | 39 months | no | yes |
| 2 | 48 | female | LUL | 13.4 mm | 4.7  mm | 0.35 | 2.37 | lobectomy | pT1a, pN1, L0, V0, Pl0 | 1 | 10L | | acinar | | yes | 66 months | no | no |

Legend: LLL, left lower lobe; LUL, left upper lobe; CTR, consolidation-to-tumor ratio; SUV, standardized uptake value
